# Data on carotid intima-media thickness and lipoprotein subclasses in type 1 diabetes from the Diabetes Control and Complications Trial and the Epidemiology of Diabetes Interventions and Complications (DCCT/EDIC)

**DOI:** 10.1016/j.dib.2015.11.036

**Published:** 2015-11-22

**Authors:** Arpita Basu, Alicia J. Jenkins, Ying Zhang, Julie A. Stoner, Richard L. Klein, Maria F. Lopes-Virella, W. Timothy Garvey, Timothy J. Lyons, The DCCT/EDIC Research Group

**Affiliations:** a Department of Nutritional Sciences, Oklahoma State University, Stillwater, OK, USA; b Section of Endocrinology & Diabetes, University of Oklahoma Health Sciences Center, Oklahoma City, OK, USA; c University of Sydney, NHMRC Clinical Trials Centre, Camperdown, Sydney, NSW, Australia; d Department of Biostatistics and Epidemiology, University of Oklahoma Health Sciences Center, Oklahoma City, OK, USA; e Division of Endocrinology, Medical University of South Carolina, Charleston, SC, USA; f The Ralph H Johnson Veterans Affairs Medical Center, Charleston, SC, USA; g Department of Nutrition Sciences, University of Alabama at Birmingham, Birmingham, AL, USA; h Centre for Experimental Medicine, Queen’s University of Belfast, Belfast, UK; i Box NDIC/EDIC, Bethesda, MD 20892, USA

**Keywords:** Lipids and lipoproteins, Carotid intima-media thickness, Type 1 diabetes, AER, Albumin Excretion Rate, BMI, Body Mass Index, CVD, CardioVascular Disease, DCCT, Diabetes Control and Complications Trial, EDIC, Epidemiology of Diabetes Interventions and Complications, IMT, Intima-Media Thickness, NMR-LSP, Nuclear Magnetic Resonance-determined Lipoprotein Subclass Profiles, T1DM, Type 1 Diabetes Mellitus.

## Abstract

Type 1 diabetes (T1DM) is associated with increased risk of macrovascular complications. We examined longitudinal associations of serum conventional lipids and nuclear magnetic resonance (NMR)-determined lipoprotein subclasses with carotid intima-media thickness (IMT) in adults with T1DM (*n*=455) enrolled in the Diabetes Control and Complications Trial (DCCT). Data on serum lipids and lipoproteins were collected at DCCT baseline (1983–89) and were correlated with common and internal carotid IMT determined by ultrasonography during the observational follow-up of the DCCT, the Epidemiology of Diabetes Interventions and Complications (EDIC) study, at EDIC ‘Year 1’ (199–1996) and EDIC ‘Year 6’ (1998–2000). This article contains data on the associations of DCCT baseline lipoprotein profiles (NMR-based VLDL & chylomicrons, IDL/LDL and HDL subclasses and ‘conventional’ total, LDL-, HDL-, non-HDL-cholesterol and triglycerides) with carotid IMT at EDIC Years 1 and 6, stratified by gender. The data are supplemental to our original research article describing detailed associations of DCCT baseline lipids and lipoprotein profiles with EDIC Year 12 carotid IMT (Basu et al. in press) [1].

**Specifications Table**

**Table:** 

Subject area	Biology
More specific subject area	Endocrinology, Lipid metabolism
Type of data	Tables
How data was acquired	NMR, Ultrasound reading
Data format	Analyzed
Experimental factors	EDIC Years 1 and 6 after intensive or conventional diabetes management in DCCT vs DCCT baseline
Experimental features	Lipoprotein subclasses and conventional lipids at DCCT baseline correlated with carotid IMT in follow-up years
Data source location	DCCT/EDIC centers (USA, Canada)
Data accessibility	Within the Data in Brief article

**Value of the data**

• Previously unreported longitudinal associations of baseline lipoprotein subclasses with carotid IMT at two follow-up time-points in a large well-characterized cohort of people with T1DM.
• May stimulate further research on the clinical utility of lipoprotein subclasses as prognostic markers for cardiovascular disease in diabetes.
• May facilitate new therapies to target lipids and lipoprotein classes significantly associated with vascular events in diabetes.

## 1 Data

The Diabetes Control and Complications Trial (DCCT) examined the effects of intensive diabetes management on the development and progression of diabetic retinopathy [2]. The cohort comprised 1441 T1DM patients aged 13–39 years who were free of overt cardiovascular disease (CVD) at enrollment in 1983–89. In 1994, the Epidemiology of Diabetes Interventions and Complications Trial (EDIC), a longitudinal observational phase of DCCT was initiated to assess the long-term effects of the DCCT intervention on cardiovascular and related complications [3]. Carotid IMT data were obtained at EDIC ‘Years’ 1 (1994–1996), 6 (1998–2000), and 12 (2004–2006). In addition to conventional lipids, nuclear magnetic resonance (NMR)-determined lipoprotein subclasses have been predictive of CVD in some T1DM studies [4], [5], [6], and were analyzed in this cohort to examine associations with a surrogate of CVD: common and internal carotid IMT.

The baseline characteristics of the participants have been described previously [1]. Table 1 shows data on the associations of DCCT baseline standard lipid profiles and NMRLSP with common carotid IMT in men and women at EDIC Years 1 and 6. Generally the data reveal positive associations of conventional LDL-cholesterol and NMR-based total IDL/LDL with IMT, and inverse associations of NMR-based large and medium VLDL subclasses, medium HDL and HDL particle size with IMT, most marked in men.

Table 2 shows data on the associations of DCCT baseline standard lipid profiles and NMR-LSP with internal carotid IMT in men and women at EDIC Years 1 and 6. Generally there are positive associations of conventional total, LDL- and non-HDL cholesterol levels with IMT in both men and women, and of NMR-based total IDL/LDL, small LDL and HDL (small)-subclasses with IMT in men only. The National Institute of Diabetes and Digestive and Kidney Diseases (NIDDK) provides access to the raw data from the DCCT/EDIC study through the NIDDK Central Repository (https://www.niddkrepository.org/home/). This website includes information regarding the data sets that are available, and details of the data request, review, and approval process.

## 2 Experimental design, materials and methods

### 2.1 Study subjects

The original DCCT cohort comprised 1441 T1DM participants aged 13–39 years at study entry (1983–1989). They had no dyslipidemia or hypertension and were randomly assigned to conventional (*n*=730) or intensive (*n*=711) diabetes treatment [2]. The data included in this article are derived from a sub-set of DCCT participants with available common and internal carotid IMT measurements at EDIC Years 1 and 6. The study was approved by the Institutional Review Boards of MUSC, University of Oklahoma Health Sciences Center (OUHSC), and all participating DCCT/EDIC centers, and written informed consent was obtained from all subjects.

### 2.2 Ultrasonography and image analysis

Common and internal carotid IMT measurements in EDIC have previously been described in detail [7]. Reliability measures for IMT readers at EDIC Years 1 and 6 have been reported previously [8]. In the current sub-study, we examined the longitudinal associations between lipoprotein profiles at DCCT entry (1983–89) and common and internal carotid IMT at EDIC Years 1 and 6.

### 2.3 NMR Lipoprotein subclass analysis

NMR-LSP was determined in first-thaw serum specimens (250 µL) using a 400-MHz proton NMR analyzer at LipoScience Inc. (Raleigh, NC, USA) as described [9]. Lipoprotein subclasses were expressed as molar particle concentrations and defined by particle diameter: VLDL subclasses (large: 60–200 nm; medium: 35–59 nm; small: 27–34 nm), intermediate density lipoprotein (IDL) (23–37 nm), LDL subclasses (large: 21.3–23 nm; small: 18.3–21.2 nm), HDL subclasses (large: 8.9–13 nm; medium: 8.3–8.8 nm; small: 7.3–8.2 nm). Average VLDL, LDL, and HDL particle sizes (nm) were determined by weighting the relative mass percentage of each subclass by its diameter.

### 2.4 DCCT baseline conventional lipid profiles, HbA_1c_, and other clinical measurements

Total cholesterol, triglyceride, and HDL-C levels were determined using previously reported enzymatic methods [10]. LDL-C was estimated according to the Friedewald equation. HbA_1c_ was measured by high-performance ion exchange liquid chromatography [11].

### 2.5 Statistical analysis

Multiple regression analyses were performed to examine correlations of conventional lipids and NMR-LSP at DCCT baseline with common and internal carotid IMT at EDIC Years 1 and 6, stratified by gender. Each lipid/lipoprotein measure was included as an independent variable in the linear model simultaneous with a fixed group of covariates that were measured at DCCT baseline: diabetes duration, smoking (yes/no), DCCT treatment group, body mass index (BMI), urinary albumin excretion rate (AER), and HbA_1c_, statin use and ultrasound imaging device. Two-tailed *p*<0.05 was considered to be statistically significant. Data were analyzed using SAS/STAT software (Version 9.2; SAS Institute Inc., Cary, NC).

## Figures and Tables

**Table 1:** DCCT baseline lipoprotein profiles vs. common carotid IMT-EDIC Years 1 and 6 (standardized linear regression coefficients^a^).

Variables	**Men (*n*=244)**				**Women (*n*=208)**			
**Year 1**		**Year 6**		**Year 1**		**Year 6**	
**NMR subclasses**	Slope (SE)	*P*	Slope (SE)	*P*	Slope (SE)	*P*	Slope (SE)	*P*
**Total VLDL and chylomicrons (nmol/L)**	0.02 (0.06)	0.74	−0.04 (0.05)	0.42	−0.05 (0.07)	0.43	−0.08 (0.07)	0.22
Large VLDL and chylomicrons (nmol/L)	−0.12 (0.06)	**0.04**	−0.04 (0.05)	0.48	0.01 (0.07)	0.9	−0.1 (0.07)	0.14
Medium VLDL (nmol/L)	−0.13 (0.05)	**0.02**	−0.05 (0.05)	0.35	−0.04 (0.07)	0.63	−0.02 (0.08)	0.78
Small VLDL (nmol/L)	0.11 (0.06)	**0.049**	−0.02 (0.05)	0.64	−0.05 (0.06)	0.43	−0.08 (0.06)	0.22
**Total IDL/LDL (nmol/L)**	0.12 (0.06)	**0.04**	0.15 (0.05)	**0.005**	0.12 (0.06)	**0.04**	0.07 (0.07)	0.33
IDL (nmol/L)	−0.06 (0.06)	0.30	−0.04 (0.05)	0.42	0.06 (0.06)	0.32	0.06 (0.06)	0.36
Large LDL (nmol/L)	0.04 (0.06)	0.48	0.06 (0.05)	0.24	0.14 (0.06)	**0.03**	0.04 (0.07)	0.57
Small LDL (nmol/L)	0.09 (0.05)	0.1	0.1 (0.05)	0.06	−0.09 (0.07)	0.17	0.01 (0.07)	0.90
**Total HDL (µmol/L)**	−0.08 (0.06)	0.20	−0.01 (0.06)	0.87	0.10 (0.06)	0.08	0.02 (0.06)	0.74
Large HDL (µmol/L)	−0.11 (0.06)	0.08	−0.05 (0.06)	0.45	0.16 (0.06)	**0.01**	0.05 (0.07)	0.46
Medium HDL (µmol/L)	−0.15 (0.07)	**0.02**	−0.03 (0.06)	0.66	0.004 (0.05)	0.94	−0.03 (0.05)	0.62
Small HDL (µmol/L)	0.1 (0.06)	0.09	0.04 (0.06)	0.52	0.04 (0.06)	0.55	0.03 (0.06)	0.59
**NMR particle diameter**								
VLDL particle size (nm)	−0.18(0.06)	**0.001**	−0.03 (0.05)	0.64	0.05 (0.07)	0.51	−0.001 (0.07)	0.98
LDL particle size (nm)	−0.05 (0.05)	0.39	−0.04 (0.05)	0.46	0.06 (0.07)	0.41	0.01 (0.07)	0.85
HDL particle size (nm)	−0.15(0.06)	**0.01**	−0.10 (0.06)	0.08	0.14 (0.07)	**0.04**	0.06 (0.07)	0.36
**Conventional lipids**								
Total cholesterol (mg/dl)	0.05 (0.06)	0.41	0.08 (0.05)	0.13	0.06 (0.06)	0.34	0.03 (0.07)	0.63
Triglyceride (mg/dl)	−0.07 (0.05)	0.14	0.07 (0.05)	0.11	−0.15 (0.09)	0.08	−0.15 (0.09)	0.11
LDL–cholesterol (mg/dl)	0.11 (0.06)	**0.04**	0.10 (0.05)	**0.049**	0.01 (0.07)	0.90	0.02 (0.07)	0.76
Non–HDL–cholesterol (mg/dl)	0.07 (0.06)	0.22	0.09 (0.05)	0.09	−0.02 (0.07)	0.75	−0.001 (0.07)	0.92
HDL–cholesterol (mg/dl)	−0.07 (0.07)	0.30	−0.02 (0.06)	0.72	0.18 (0.06)	**0.003**	0.08 (0.06)	0.18

*P* values in bold if significant (<0.05).

a Models were adjusted for DCCT randomization, albumin excretion rate, HbA_1C_, diabetes duration, body mass index, and current smoking at DCCT baseline (1983–89). The regression models were also adjusted for statin use (any time during the DCCT baseline to EDIC year 12), ultrasound devices, and image readers at EDIC year 1 and 6 respectively.

**Table 2:** DCCT baseline lipoprotein profiles vs. internal carotid IMT-EDIC Years 1 and 6 (standardized linear regression coefficients^a^).

Variables	**Men (*n*=242)**				**Women (*n*=203)**			
**Year 1**		**Year 6**		**Year 1**		**Year 6**	
**NMR subclasses**	Slope (SE)	*P*	Slope (SE)	*P*	Slope (SE)	*P*	Slope (SE)	*P*
**Total VLDL and chylomicrons (nmol/L)**	−0.01 (0.07)	0.92	−0.03 (0.07)	0.67	0.01 (0.06)	0.83	0.06 (0.07)	0.35
Large VLDL and chylomicrons (nmol/L)	−0.04 (0.07)	0.55	−0.07 (0.07)	0.33	−0.003 (0.07)	0.97	0.05 (0.07)	0.50
Medium VLDL (nmol/L)	0.07 (0.07)	0.28	0.02 (0.07)	0.80	0.04 (0.07)	0.57	0.01 (0.07)	0.84
Small VLDL (nmol/L)	−0.05 (0.07)	0.51	−0.04 (0.07)	0.55	0.001 (0.06)	0.99	0.06 (0.06)	0.33
**Total IDL/LDL (nmol/L)**	0.23 (0.07)	**0.002**	0.14 (0.07)	0.06	0.004 (0.06)	0.95	−0.02 (0.06)	0.74
IDL (nmol/L)	0.09 (0.07)	0.22	0.004 (0.07)	0.96	0.08 (0.06)	0.18	0.09 (0.06)	0.13
Large LDL (nmol/L)	0.02 (0.07)	0.75	0.03 (0.07)	0.61	−0.002 (0.06)	0.97	−0.04 (0.06)	0.52
Small LDL (nmol/L)	0.14 (0.07)	**0.04**	0.09 (0.07)	0.21	−0.03 (0.07)	0.65	−0.03 (0.07)	0.65
**Total HDL (µmol/L)**	0.09 (0.08)	0.26	0.07 (0.08)	0.38	−0.02 (0.05)	0.67	−0.09 (0.06)	0.12
Large HDL (µmol/L)	0.04 (0.08)	0.63	−0.07 (0.08)	0.42	−0.03 (0.06)	0.62	−0.03 (0.06)	0.56
Medium HDL (µmol/L)	0.10 (0.08)	0.23	0.02 (0.08)	0.79	−0.03 (0.05)	0.52	−0.06 (0.05)	0.23
Small HDL (µmol/L)	0.20 (0.08)	**0.01**	0.09 (0.08)	0.26	0.001 (0.06)	0.99	−0.001 (0.06)	0.99
**NMR particle diameter**								
VLDL particle size (nm)	−0.0001 (0.07)	1.00	−0.06 (0.07)	0.44	0.03 (0.07)	0.66	0.02 (0.07)	0.75
LDL particle size (nm)	−0.08 (0.07)	0.25	−0.03 (0.07)	0.63	0.02 (0.07)	0.75	0.02 (0.06)	0.71
HDL particle size (nm)	−0.13 (0.08)	0.08	0.06 (0.07)	0.35	0.07 (0.06)	0.29	−0.12 (0.08)	0.11
**Conventional lipids**								
Total cholesterol (mg/dl)	0.17 (0.07)	**0.02**	0.13 (0.07)	0.07	0.15 (0.06)	**0.01**	0.05 (0.06)	0.44
Triglyceride (mg/dl)	0.02 (0.06)	0.76	0.04 (0.06)	0.51	0.04 (0.09)	0.64	0.06 (0.09)	0.52
LDL–cholesterol (mg/dl)	0.17 (0.07)	**0.02**	0.14 (0.07)	**0.05**	0.12 (0.06)	**0.05**	0.04 (0.06)	0.51
Non–HDL–cholesterol (mg/dl)	0.16 (0.07)	**0.03**	0.12 (0.07)	0.09	0.12 (0.06)	**0.05**	0.05 (0.07)	0.45
HDL–cholesterol (mg/dl)	0.05 (0.08)	0.57	0.04 (0.08)	0.62	0.09 (0.06)	0.12	0.01 (0.06)	0.87

*P* values in bold if significant (<0.05).

a Models were adjusted for DCCT randomization, albumin excretion rate, HbA1C, diabetes duration, body mass index, and current smoking at DCCT baseline (1983–89). The regression models were also adjusted for statin use (any time during the DCCT baseline to EDIC year 12), ultrasound devices, and image readers at EDIC year 1 and 6 respectively.
